# Th17/Treg ratio derived using DNA methylation analysis discriminates allergen-induced early from dual asthmatic responses

**DOI:** 10.1186/1710-1492-10-S1-A46

**Published:** 2014-03-03

**Authors:** Amrit Singh, Masatsugu Yamamoto, Jian Ruan, Jung Young Choi, Gail M Gauvreau, Paul M O'Byrne, Sven Olek, Ulrich Hoffmueller, Christopher Carlsten, J Mark FitzGerald, Louis-Philippe Boulet, Scott J Tebbutt

**Affiliations:** 1 James Hogg Research Centre, St. Paul’s Hospital, University of British Columbia, Vancouver, British Columbia, V6Z 1Y6, Canada; 2 Department of Medicine, McMaster University, Hamilton, Ontario, L8S 4L8, Canada; 3 Epiontis GmbH, Berlin, Germany; 4 Vancouver Coastal Health Research Institute, Vancouver General Hospital, Vancouver, British Columbia, V5Z 1M9, Canada; 5 Centre de Pneumologie de L’Hopital, Université Laval, Sainte-Foy, Quebec, G1V 0B4, Canada

## Background

Atopic allergic asthmatic individuals experience acute bronchoconstriction (early response) upon allergen exposure. Several hours after the initial exposure, some individuals exhibit a chronic late phase (dual responders, DRs) whereas others do not (early responders, ERs). The purpose of this study is to determine changes in Th17 and regulatory T (Treg) cell numbers and their associated gene expression profiles in whole blood between allergen-induced ERs and DRs.

## Methods

14 participants with mild, atopic asthma (8 ERs and 6 DRs) underwent a cat allergen inhalation challenge as part of the AllerGen Clinical Investigator Collaborative. Whole blood was collected immediately prior to challenge (pre) and 2 hours post-challenge. DNA methylation analysis was used to measure the frequency of Th17, Treg, B and T cells (Epiontis, Germany). Whole blood transcriptome profiling was performed using Affymetrix GeneChip® Human Gene 1.0 ST Arrays. Statistical analysis was performed using R.

## Results

Sum of the T cell and B cell frequencies obtained using the methylation assays strongly correlated (r = 0.95) with the lymphocyte frequency obtained using a hematolyzer. Allergen inhalation did not significantly (p>0.05) change Th17, Treg, B and T cell counts between ERs and DRs. However, the Th17/Treg ratio was significantly (p=0.03) different between ERs and DRs post challenge. 199 genes positively correlated with Th17 cells at an FDR of 5%. 463 genes positively correlated with Treg cells at an FDR of 5%. Th17 genes were inversely correlated with Treg genes.

## Conclusions

Th17/Treg ratio derived using DNA methylation analysis discriminates allergen-induced early from dual asthmatic responses. The inverse correlation between Th17 genes and Treg genes may be indicative of the inflammatory or suppressive phenotypes of these cells.

